# Strong Inference in Mathematical Modeling: A Method for Robust Science in the Twenty-First Century

**DOI:** 10.3389/fmicb.2016.01131

**Published:** 2016-07-22

**Authors:** Vitaly V. Ganusov

**Affiliations:** ^1^Department of Microbiology, University of TennesseeKnoxville, TN, USA; ^2^Department of Mathematics, University of TennesseeKnoxville, TN, USA; ^3^National Institute for Mathematical and Biological Synthesis, University of TennesseeKnoxville, TN, USA

**Keywords:** robust science, mathematical modeling, immunology, microbiology, public health, scientific method

## Abstract

While there are many opinions on what mathematical modeling in biology is, in essence, modeling is a mathematical tool, like a microscope, which allows consequences to logically follow from a set of assumptions. Only when this tool is applied appropriately, as microscope is used to look at small items, it may allow to understand importance of specific mechanisms/assumptions in biological processes. Mathematical modeling can be less useful or even misleading if used inappropriately, for example, when a microscope is used to study stars. According to some philosophers (Oreskes et al., 1994), the best use of mathematical models is not when a model is used to confirm a hypothesis but rather when a model shows inconsistency of the model (defined by a specific set of assumptions) and data. Following the principle of strong inference for experimental sciences proposed by Platt (1964), I suggest “strong inference in mathematical modeling” as an effective and robust way of using mathematical modeling to understand mechanisms driving dynamics of biological systems. The major steps of strong inference in mathematical modeling are (1) to develop multiple alternative models for the phenomenon in question; (2) to compare the models with available experimental data and to determine which of the models are *not* consistent with the data; (3) to determine reasons why rejected models failed to explain the data, and (4) to suggest experiments which would allow to discriminate between remaining alternative models. The use of strong inference is likely to provide better robustness of predictions of mathematical models and it should be strongly encouraged in mathematical modeling-based publications in the Twenty-First century.

## 1. The core of mathematical modeling

What is the use of mathematical modeling in biology? The answer likely depends on the background of the responder as mathematicians or physicists may have a different answer than biologists, and the answer may also depend on the researcher's definition of a “model.” In some cases models are useful for estimation of parameters underlying biological processes when such parameters are not directly measurable. For example, by measuring the number of T lymphocytes over time and by utilizing a simple model, assuming exponential growth, we can estimate the rate of expansion of T cell populations (De Boer et al., 2001). In other cases, making the model may help think more carefully about contribution of multiple players and their interactions in the observed phenomenon. In general, however, mathematical models are most useful when they provide important insights into underlying biological mechanisms. In this opinion article, I would to provide my personal thoughts on the current state and future of mathematical modeling in biology with the focus on the dynamics of infectious diseases. As a disclosure I must admit that I am taking an extreme, provocative view, based on personal experience as a reader and a reviewer. I hope that this work will generate the much needed discussion on uses and misuses of mathematical models in biology and perhaps will result in quantitative data on this topic.

In my experience, in the area of dynamical systems/models of the within-host and between-host dynamics of infectious diseases, the two most commonly given answers to the question of the “use of mathematical models” are (1) models help us understand biology better; and (2) models help us predict the impact of interventions (e.g., gene knockouts/knockins, cell depletions, vaccines, treatments) on the population dynamics. Although there is some truth to these answers the way mathematical modeling in biology is generally taught and applied rarely allows one to better understand biology. In some cases mathematical models generate predictions which are difficult or impossible to test, the latter making such models unscientific per the definition of a scientific theory according to one of the major philosophers of science in the Twentieth Century Karl Popper (Popper, 2002). Moreover, mathematical modeling may result in questionable recommendations for public health-related policies. My main thesis is that while, in my experience, much of current research in mathematical biology is aimed at finding the right model for a given biological system, we should pay more attention to understanding which biologically reasonable models do not work, i.e., are not able to describe the biological phenomenon in question. According to Karl Popper, proving a given hypothesis to be correct is impossible while rejecting hypotheses is feasible (Oreskes et al., 1994; Popper, 2002).

What is a mathematical model? In essence, mathematical model is a hypothesis regarding a phenomenon in question. While any specific model always has an underlying hypothesis (or in some cases, a set of hypotheses), the converse is not true as multiple mathematical models could be formulated for a given hypothesis. In this essay I will use words “hypothesis” and “model” interchangeably. The core of a mathematical model is the set of model assumptions. These assumptions could be based on some experimental observations or simply be a logical thought based on everyday experience. For example, for an ordinary differential equation (ODE)-based model, the assumptions are the formulated equations which include functional terms of interactions between species in the model, parameters associated with these functions, and initial conditions of the model. The utility of mathematics lies in our ability to logically follow from the assumptions to conclusions on the system's dynamics. Thus, mathematical modeling is a logical path from a set of assumptions to conclusions. Such a logical path from axioms to theorems was termed by some as a mathematical revolution in the Twentieth Century (Quinn, 2012). However, while in mathematics it is vital to formulate a complete set of axioms/assumptions to establish verifiable, true statements such as theorems (Quinn, 2012), a complete set of assumptions is impossible in any biology-based mathematical model due to the openness of biological systems (or any other natural system, Oreskes et al., 1994). Therefore, biological conclusions stemming from analysis of mathematical models are inherently incomplete and are in general strongly dependent on the assumptions of the model (De Boer, 2012). While such dependency of model conclusions on model assumptions may be viewed as a weakness but it is instead the most significant strength of mathematical modeling! By varying model assumptions one can vary model predictions and subsequently by comparing predictions to experimental observations, sets of assumptions which generate predictions consistent and inconsistent with the data can be identified. This is the **core of mathematical modeling** which can provide profound insights into biological processes. While it is often possible to provide mechanistic explanations for some biological phenomena from intuition—and many biologists do it—it is often hard to identify sets of implicit assumptions made during such a verbal process. Mathematical modeling by requiring one to define the model specifies such assumptions explicitly. Inherent to this interpretation of mathematical modeling is the need to consider multiple sets of assumptions (or models) to determine which are consistent and, more importantly, which are not consistent with experimental observations. Rather than a thorough expedition to test multiple alternative models, in my experience as a reader and a reviewer many studies utilizing mathematical modeling in biology have been a quest to find (and analyze) a single “correct” model.

I would argue that studies in which a single model was considered and in which the developed model was not rigorously tested against experimental data, do not provide robust biological insights (see below). Pure mathematical analysis of the model and its behavior (e.g., often performed steady state stability analyses for ODE-based models) often provides little insight into the mechanisms driving dynamics in specific biological systems. Failure to consider alternative models often results in biased interpretation of biological observations. Let me give two examples.

Discussion of predator-prey interactions in ecology often starts with the Lotka-Volterra model which is built on very simple and yet powerful basic assumptions (Mooney and Swift, 1999; Kot, 2001). The dynamics of the model can be understood analytically and predictions on the dynamics of predator and prey abundances can be easily generated. The observation of the hares and lynx dynamics in Canada has been often presented as evidence that predator-prey interactions driven the dynamics of this biological system (Mooney and Swift, 1999). While it is possible that the dynamics was driven by predator-prey interactions, recent studies also suggest that the dynamics could be driven by self-regulating factors and weather activities influencing independently each of the species (Brauer and Castillo-Chávez, 2001; Zhang et al., 2007). A more robust modeling approach would be to start with observations of lynx and hare dynamics and ask about biological mechanisms which could be driving such dynamics including predator-prey interactions, seasonality, or both (Hilborn and Mangel, 1997). The data can then be used to test which of these sets of assumptions is more consistent with experimental data using standard model selection tools (Burnham and Anderson, 2002).

In immunology, viral infections often lead to generation of a large population of virus-specific effector CD8 T cells, and following clearance of the infection, there is formation of memory CD8 T cells (Ahmed and Gray, 1996; Kaech and Cui, 2012). However, how memory CD8 T cells are formed during the infection has been a subject of a debate (Ahmed and Gray, 1996). One of the earlier models assumed that memory precursors proliferate during the infection and produce terminally differentiated, nondividing effector T cells, which then die following clearance of the infection (Wodarz et al., 2000; Bocharov et al., 2001; Wodarz and Nowak, 2002; Fearon et al., 2006). While this model was used to explain several biological phenomena, later studies have shown that this model failed to accurately explain experimental data on the dynamics of CD8 T cell response to lymphocytic choriomengitis virus (Antia et al., 2005; Ganusov, 2007). More precisely, the model was able to accurately fit experimental data but it required unphysiologically rapid interdivision time for activated CD8 T cells [e.g., 25 min in Ganusov (2007)] which was inconsistent with other measurements made to date. Constraining the interdivision time to a larger value (e.g., 3 h) resulted in a poor model fit of the data. Therefore, development of adequate mathematical models cannot be all based on “basic principles” and must include comparison with quantitative experimental data.

These examples illustrate how mathematical modeling can teach us about mechanisms underlying biological processes. When a model is developed using some basic biological assumptions/mechanisms and yet such a model is unable to accurately describe quantitative biological data, we learn something. We learn that the mechanisms that we thought should be important in explaining the phenomenon are incorrect (or that we modeled them incorrectly). In this case, modeling provides important information that some aspects of biology that we thought we knew we actually do not know. In the case of memory CD8 T cell differentiation, the poor assumption was that effector T cells do not proliferate (Ganusov, 2007). An alternative situation is when it is believed that only one mechanism explains a biological phenomenon, and yet several different models can be formulated and all models are able to accurately describe experimental data. Again, such a result would illustrate that specific data can be explained by more than one mechanism and additional experiments are needed to further discriminate between alternative models. Although this has not been formally done, two alternative mechanisms (predator-prey and seasonality) may be reasonable explanations of the hare-lynx dynamics in Canada.

## 2. Strong inference in mathematical modeling

Strong inference was proposed over 50 years ago to promote rapid science (Platt, 1964). Platt suggested that despite a commonly spread “…polite fiction that all science is equal…some areas of science progress faster than others” (Platt, 1964). Platt (1964) proposed that by choosing well formulated questions and hypotheses and by designing discriminatory experiments, one can progress faster with understanding of the underlying phenomena. According to strong inference, the following steps must be taken to investigate a given scientific question (Platt, 1964):

1. Devising alternative hypotheses;
2. Devising a crucial experiment (or several of them), with alternative possible outcomes, each of which will, as nearly as possible, exclude one or more of the hypotheses;
3. Carrying out the experiment so as to get a clean result;
1'. Recycling the procedure, making subhypotheses or sequential hypotheses to refine the possibilities that remain; and so on.

These recommendations were highly influential as judged by the number of citation (1439 in Web of Science or 2867 in Google scholar as of April 5th, 2016); however, it does not appear that they have been widely adopted in biological sciences (Jewett, 2005). Two major points of these recommendations include (1) formulation of a set of alternative hypotheses and (2) attempt to reject, not to confirm, these hypotheses. The idea of formulating multiple hypotheses goes back to another important paper on “The method of multiple working hypotheses” (Chamberlin, 1890) which recently received an update (Elliott and Brook, 2007). The idea of testing hypotheses to reject them goes back to Karl Popper, who proposed that falsification of hypotheses is the core of the scientific method (Popper, 2002). Strong inference received its share of criticism suggesting that it cannot be applied in some areas of research and that it does not promote rapid science (O'Donohue and Buchanan, 2001). Indeed, testing *n* > 1 multiple hypotheses is unlikely to provide rapid progress because it would probably take *n* times longer to find the answer as compared to that if there were only one hypothesis to start with. However, strong inference will likely result in more robust results than results based on a single hypothesis, and therefore, overall, multiple hypotheses-driven research provides more rapid progress for the field as it cuts out early wrong leads. One author suggested that the use of strong inference may occur more frequently in industry than in academia due to a higher focus of industrial research on robustness rather than novelty (Ehlers, 2016). Robust conclusions rather than novel results are also viewed as a feature of good scientists both by general public and professional researchers (Ebersole et al., 2016).

In my view, not all mathematical modeling studies are equal and some provide better insights into biological mechanisms than others. By extending Platt's ideas to mathematical modeling I propose the following steps for “strong inference in mathematical modeling” in biology:

1. For a given biological question and associated experimental data, formulate several alternative mathematical models aimed at explaining the data;
2. Compare model predictions with experimental data with the goal of excluding as many of the alternative models as possible;
3. For the rejected models, determine reasons why the models were not able to accurately describe the data;
4. For the models that are consistent with the data, generate predictions for experiments which would allow one to discriminate between these alternative models;
1'. As new data are available, recycle the procedure by making sub-models, alternative models, and so on.

To avoid misinterpretation two issues must be explained further: what different models are and what it means to reject a model.

There are two levels at which alternative models can be defined. One is the basic/core mechanism of the mathematical model and another is specific model formulations within such a core mechanism. Using hare-lynx dynamics as an example, two core mechanisms could include predator-prey interactions or season-driven dynamics. (Perhaps the reader already came up with a third core mechanism?) Using a given specific core mechanism one now can write different formulations of the model, for example, how predator consumes the prey and how the prey biomass translates into predator biomass. Multiple formulations are possible and these all are alternative models, and yet they all have the same basic core mechanism. In essence, the model core is an equivalent of the main hypothesis responsible for the observed phenomenon. Similarly, seasonality can enter the model directly assuming time-dependent birth/death rates of hares and lynx or indirectly by assuming time-dependent variability in resources. These formulations also can be viewed as alternative models. Rejection of a specific mathematical model does not necessarily invalidate the core mechanism but rejection of a set of alternative models based on a given core mechanism will raise doubts whether such a core mechanism is responsible for the observed phenomenon. The best use of strong inference is a rejection of a core mechanism.

Criteria of model rejection are not well established and rejection can be done on absolute or relative grounds. When comparing model predictions and data one could ask if the model is adequately describing the data. Two tests could be of particular importance such as goodness of fit test and lack of fit test (Bates and Watts, 1988). These tests require data with sufficient richness but in some cases, incompatibility between model and data can be determined (Noecker et al., 2015). When using a set of alternative models other tests such as likelihood ratio test or information criteria (AIC, BIC, etc.) can be also used (Bates and Watts, 1988; Burnham and Anderson, 2002; Johnson and Omland, 2004) to determine which of the models are less likely to be consistent with the data. Similarly, comparison with data may allow to reject a core mechanism or more commonly, reject specific formulations of the core mechanism. Issues associated with identifiability of mathematical models and precise estimation of model parameters in some case may not allow to reject specific models (Meshkat et al., 2009; Raue et al., 2009).

Proper application of strong inference in mathematical modeling depends critically on choosing a “good” question which has only a limited number of possible core mechanisms. It is clear that “big” fundamental questions often have many potential answers (O'Donohue and Buchanan, 2001) and from the perspective of strong inference, big questions can rarely be exhaustively explored. As continuous application of the method of multiple working hypotheses “develops a habit of parallel or complex thought” (Chamberlin, 1890), continuous application of strong inference allows development of a skill of asking the “good” questions and recognition when asked questions are “bad.”

As the method of multiple working hypotheses has a “danger of vacillation” (Chamberlin, 1890), strong inference may fail when none of the alternative models can be rejected. In fact, it has been argued that inability to reject hypotheses/models may be a feature of ecological studies (Hobbs and Hilborn, 2006). One proposed solution is to use model averaging where predictions of different models are “weighted” based on the models' consistency with experimental data (Hoeting et al., 1999; Burnham and Anderson, 2002). Model averaging is not without problems, however, including situations where alternative models generate contradictory predictions (Grueber et al., 2011). In my view, inability to apply principles of strong inference to reject some of the alternative models indicates two potential problems: (1) the data are poor and insufficient to discriminate between alternative models (so more and better data need to be collected), and (2) the formulated question is “bad” (so a better formulated question is needed).

One useful example of the use of strong inference comes from the analysis of movement patterns of activated CD8 T cells in murine brains (Harris et al., 2012). Using intravital imaging the authors recorded coordinates of T cells in the brain over long periods of time. By comparing predictions of multiple mathematical models the authors concluded that only one in the list of several alternative models, based on generalized Levy walks, could explain all data with reasonable quality (Harris et al., 2012). Future studies utilizing further strong inference would need to discriminate between cell-intrinsic vs. environment-driven core mechanisms explaining this type of walk of T cells in the brain.

With principles of strong inference the power of mathematical modeling can be truly revealing. Closer collaborations between experimentalists and modelers leading to discrimination between alternative models using data would likely result in substantial robust gains in our understanding of biological processes.

## 3. Dangers of single hypothesis/model-driven research

While scientific benefits of multiple hypotheses/models-driven research are hard to deny, dangers of using single hypotheses in research have not been widely emphasized. Already in 1890, Chamberlin (1890) warned about biases resulting from “dominant theory” or “single hypothesis”-driven research and why thinking in terms of multiple hypotheses must extend beyond science and be common practice for everyone in the world. I would like to present three examples, in which single hypothesis/mathematical model-driven research limits and sometimes biases our understanding of biology. These examples represent my hypothesis on limited robustness of single mathematical model-based studies; this hypothesis will have to be tested and perhaps rejected in the future.

### 3.1. Biased predictions

One of the virtues of mathematical models is often cited their predictive power. Indeed, mathematical models are used to make predictions in many areas of science including biology. The types of models used to make predictions vary in their complexity from simple, few equations-based models to models including hundreds of variables. How robust are predictions of such models? My thesis is that predictions based on a single mathematical model are unlikely to be robust (De Boer, 2012).

Recently, Evans et al. (2013) questioned whether general, very simple models are useful in making quantitative predictions on vital, public-health related issues. The authors argued that such general models by design are relatively simple and are aimed at describing as many situations as possible. The authors also argued that models that are designed for specific systems and parameterized from specific experimental data, are likely to be more precise in predictions. Such case-specific models are thought to be more useful in guiding policies for control of infectious diseases (Evans et al., 2013). The authors illustrated their point by discussing the predictions of two mathematical models on the level of vaccination required to eradicate rabies in the fox populations in Europe (Anderson et al., 1981; Eisinger and Thulke, 2008). Evans et al. (2013) argued that simple, susceptible-infected-recovered mathematical model overestimated the level of vaccination needed for rabies eradication (Anderson et al., 1981). Such a simple model predicted that 70% of foxes had to be vaccinated for efficient control. A more complex model, including details of the local spread of the infection from rabid to susceptible foxes, predicted a lower vaccination level of 60% (Eisinger and Thulke, 2008). Although such a 10% difference may appear small, Eisinger and Thulke (2008) suggested that the vaccination campaign based on the prediction of the simple model may have cost over several millions of euros more than was needed. The authors concluded that in order to make public health-related predictions for a specific biological system, the models should include sufficient detail about that system so the model predictions are accurate and precise (Evans et al., 2013). Thus, predictions of a single model may not be robust, and in some cases, predicted interventions may cost more than needed.

Another example comes from early predictions of potential size of the Ebola virus epidemics in Africa in 2014–2016 (Butler, 2014). Initial studies by considering simple models predicted devastating impact of the epidemic on human population which luckily did not occur (Butler, 2014; Pandey et al., 2014). Later analyses revealed that simple models were inadequate by ignoring potential heterogeneity in behavior which translated into large variability in transmission efficacy (Drake et al., 2015). Although there is a consensus that mathematical modeling is needed to understand biological phenomena including epidemiology of infectious diseases (Lofgren et al., 2014), non-robust model predictions which overestimate risks are perhaps even more harmful than models that underestimate the risks. In fact, good modeling practice is in general to provide minimal estimates of the risk. Examples of wrong predictions may fuel unwarranted public debate on trustworthiness of mathematical models, for example, predicting climate change. Taken together, studies that are based on the analysis of a single model are not expected to produce robust predictions (Oreskes et al., 1994). Predictive studies illustrating which alternative models have been considered in the analysis, which models have been rejected and why, and whether predictions of the remaining models are self-consistent, will lead to robust predictions and should be encouraged.

### 3.2. Unreproducible science

The great feature of science is its self-correcting nature. Some theories have persisted for decades but have been shown later to be incorrect as new ideas and data accumulated. While exceptions clearly exist and there are still common myths despite experimental evidence otherwise (Scudellari, 2015), science has been mostly self-correcting. I would argue that in some cases consideration of a single hypothesis and failure to consider and reject alternatives has caused dominance of an eventually wrong theory. In some cases, self-correction in sciences took long time with resources wasted and lives affected. One example is on the development of understanding of motions of planets with a complete dominance of Ptolemy's theory of immotile Earth with Sun and planets moving in circular orbits (Danielson and Graney, 2014). If Tycho Brahe, one of the major astronomers collecting data to support Ptolemy's circular orbits-based theory, and other scientists at the time considered alternatives of elliptic circles and movable Earth, perhaps science would progress faster, reach more robust conclusions, and Bruno and Galileo would not have suffered (Danielson and Graney, 2014). There is more recent, perhaps an extreme example of a crime conviction of an innocent person based on consideration of a single hypothesis (Nuzzo, 2015).

The common practice of considering a single hypothesis and collecting data to “prove” it can bias interpretation and may result in unreproducible results. In recent years it has been noted by several groups of investigators that many of the results in biological sciences are unreproducible (Prinz et al., 2011; Begley and Ellis, 2012; Collaboration, 2015; Freedman and Gibson, 2015; Freedman et al., 2015). In particular, biotech company Amgen attempted to reproduce 53 “landmark” papers from cancer biology and was able to reproduce only 6 (Begley and Ellis, 2012). Overall, a recent review suggests that at least 50% of reanalyzed studies are unreproducible (Freedman et al., 2015). If these findings can be extrapolated to the whole field of biomedical research one study estimates that over $28B are wasted on unreproducible studies, and half of those expenditures are suggested to result from inappropriate study design and data analysis (Freedman et al., 2015).

It remains unknown whether reproducibility of mathematical modeling-based studies is different from that of science in general (or biology in particular, Boulesteix et al., 2015). For example, one recent study could reproduce less than half of bioinformatic analyses of published microarray gene expression data (Ioannidis et al., 2009). The definition of reproducibility may be difficult in general as it may vary by researcher (Goodman et al., 2016). For one type of mathematical modeling studies which do not involve any experimental data we generally expect full reproducibility if the authors correctly wrote and analyzed their model and/or appropriately simulated its dynamics. However, programing errors may still occur. A lower level of reproducibility may be expected for studies utilizing both mathematical models and analysis of experimental data. I analyzed a subset of data from a recent survey by Nature (Baker, 2016) by focusing on responses by scientists from the field of “Biology” with expertise in “Bioinformatics and Computational Biology” (*n*_1_ = 36) or “Systems Biology” (*n*_2_ = 9, *n* = *n*_1_ + *n*_2_ = 45 surveys in total). I found that computational biologists are at least as skeptical about the state of reproducibility of studies in their fields as compared to all scientists surveyed. In particular, computational biologists believe that on average only 50% of studies in their field are reproducible (compared to 58% for general population, Mann-Whitney test, *p* = 0.02), 27% believe that computational biology has similar level of reproducibility compared to other fields (vs. 21% for all scientists, $\chi_{\left(1\right)}^{2}=0.76$, *p* = 0.38), and 73% of computational biologists believe that failure to reproduce results is the major problem in the field (as compared to 59% of all scientists surveyed, $\chi_{\left(1\right)}^{2}=3.85$, *p* = 0.05). Interestingly, 20% of computational biologists were told that someone could not reproduce their work (vs. 18% for all scientists, $\chi_{\left(1\right)}^{2}=0.12$, *p* = 0.73). Thus, there is a general concern about the level of reproducibility of mathematical modeling-based studies.

A large number of unreproducible studies is paralleled by a recent increase in percentage of retracted peer-reviewed papers (Fang et al., 2012; Grieneisen and Zhang, 2012; Fanelli, 2013; Castillo, 2014). While increased scrutiny of published papers may have contributed to the rise in the number of retracted articles (Fanelli, 2013), the increased competition in research, especially in biomedical sciences, leading to the “publish-or-perish” culture is a very like cause for the growing number of unreproducible studies and retracted papers (Steen et al., 2013). The number of retracted mathematical modeling-based papers remains relatively low (a simple search for “mathematical model” on RetractionWatch.com yielded under ten hits as of April 5th, 2016).

The need for more robust ways of doing science, including mathematical modeling, is well recognized (Begley and Ellis, 2012; Fang and Casadevall, 2012). By focusing mathematical modeling analyses on a single model and by showing qualitative consistency of the model and data we commit a cognitive/confirmation bias (Kaptchuk, 2003; Editorial, 2015). Confirmation bias appears to be widespread in the mathematical modeling literature where consistency of a model with experimental observations occurs much more frequently than rejection of models. Even in cases when model predictions match qualitatively other, potentially independent data, there is a risk of so-called “therapeutic illusion” (Casarett, 2016), an inability to recognize that alternative mechanisms, not included in the model, could explain additional data too. Several suggestions have been made to improve reproducibility and robustness of science including use of strong inference (Nuzzo, 2015), improved trainings (Moher and Altman, 2015), performing blind analyses of the data (MacCoun and Perlmutter, 2015), the need for independent analyses of the same data/models by different teams prior to publication (Silberzahn and Uhlmann, 2015), and standardization of tools (Baker, 2015). There is also a need to reduce overoptimistic reporting in mathematical modeling-based studies (Boulesteix, 2015) and reduce uncertainties in predictions of mathematical models (Kirk et al., 2015). The use of principles of strong inference should increase robustness of predictions of mathematical models and in general, should reduce the amount of unreproducible research in biology.

### 3.3. Development of large models

The formulation and analysis of multiple alternative mathematical models can clearly increase robustness of conclusions and improve our ability to make accurate predictions. Robustness of predictions of mathematical models for public health-related policies is particularly important. To avoid the need to formulate multiple alternative models for a given phenomenon researchers often construct models that include many of known mechanisms in the biological system of interest. Such a model is then expected to be able to explain a large number of different phenomena, and there is a hope that at some choice of parameters the model behavior will capture true biological forces at play. Such a model is viewed as useful to make specific predictions of the impact of interventions on population dynamics (Bru and Cardona, 2010; Cilfone et al., 2015). This trend for “systems” view on biological phenomena is becoming more popular and it is now being questioned whether simple models which include only a few major details about biological system are useful in making relevant forecasts (Evans et al., 2013). One of the major problems of large and complex models is that by including many mechanisms and details these models become as complex as phenomena they are trying to explain precluding detailed understanding of such models. Furthermore, by including multiple details such large models can rarely if ever be rejected which essentially makes them unscientific per Karl Popper (Popper, 2002; Ellis and Silk, 2014).

Large complex models are often compared to data to illustrate their plausibility. However, with tens to hundreds of parameters complex models can easily explain one or several datasets. Such model overfitting of the data should never be viewed as model confirmation (Oreskes et al., 1994). Only few parameters are needed to generate complex patterns as famous saying states: “with four parameters I can fit an elephant, and with five, I can make him wiggle his trunk” (Mayer et al., 2010; Ditlev et al., 2013). Development of large, complex models can be useful if such models show inconsistency of specific mechanisms with sets of experimental observations. Predictions of large models should be treated with caution unless it has been established which alternative models/mechanisms have been rejected during model development (Oreskes et al., 1994). Iterative process of model development, testing, and calibration using sufficiently extensive datasets may result in large mathematical models of robust predictive power; mathematical models predicting weather are one good example (Bauer et al., 2015). Yet, even well calibrated weather prediction models have reasonable accuracy only for relatively short-term predictions (Bauer et al., 2015).

## 4. Changing training in mathematical biology

Given intuitive benefits of multiple models-driven research it is perhaps strange to realize that it remains quite rare. In part this is due to widely adopted approach to find models which explain phenomena. I believe that “the approach to find the right model” starts very early in education of a mathematical biologist, probably during undergraduate or early graduate career. Many of the classical textbooks on mathematical modeling in biology have a similar theme: (1) identify a biological problem, (2) develop a mathematical model for the problem; the degree of complexity of the model should depend on the complexity of the problem and/or underlying biology, (3) analyze the model; (4) draw the conclusions from the model behaviors and extrapolate the conclusions to the actual biological system (Segel, 1984; Mooney and Swift, 1999; Kot, 2001; Ellner and Guckenheimer, 2006; Vries et al., 2006; Percus, 2012). In this approach the developed model is often treated as a very good representation of the actual biological system and rarely the basic assumptions of the model are challenged. Education in physics and engineering proceeds in a similar fashion where complex mathematical models are derived from basic principles which are accepted to be true either because of some fundamental experiments or simply because of intuition. This approach, although being relatively straightforward, fosters an impression that if one starts with a good set of assumptions this will lead to a model which should not be questioned. Experimental data are often brought as support of the model, and when the model predictions are consistent with some, often qualitative data, the model appears to be a strong reflection of the reality (Simberloff, 2007). However, rarely the basic feature of mathematical models—that predictions are the direct consequences of the model assumptions—is investigated thoroughly by identifying model assumptions which are most critical for the “consistency” between the model and experimental observations, and which assumptions would allow the model to “fail” at explaining the data. Furthermore, in many cases consistency between models and data is indicated by qualitative or semi-quantitative comparison which does not allow to investigate in a rigorous sense whether the model is indeed an accurate enough representation of the data (Jin et al., 1999; Wang et al., 2015).

While many methods are likely to improve robustness of mathematical modeling-based (and other scientific) studies, the widespread use of strong inference is likely to be important in this endeavor (Nuzzo, 2015). Design of multiple alternative models forces the researcher to deeply understand the underlying biological question and not be satisfied with standard answers that “this is well known” but to require solid experimental support for major model assumptions. *Education of future generations of students in mathematical modeling should focus more on deeper understanding of biological details and on investigating which aspects of their models could be wrong*. If we substitute “theory” with “model,” it was very nicely said by Ellis and Silk (2014) that research often “boils down to clarifying one question: what potential observational or experimental evidence is there that would persuade you that the theory is wrong and lead you to abandoning it? If there is none, it is not a scientific theory.” Finding boundaries when the model “breaks” at explaining the phenomenon in question would reveal limitations of the model and of its predictions. *Therefore, future mathematical modelers should be able to understand details of biological experiments, how the data are collected and analyzed, so such data are used with most efficiency for model development and testing. Such training thus must extend beyond traditional education in mathematics, engineering, and computer science*.

One of the major difficulties with multiple models-driven research and strong inference is to identify the number of alternative models/hypotheses one needs to consider to satisfy principles of strong inference (Platt, 1964). Choosing a “good” question is key in this process. Wise application of strong inference requires selection of “good” questions for which only a limited number of alternative hypotheses (or core mechanisms) exist (Platt, 1964). Choosing the “good” question is an endeavor and skill on its own; it is a part of scientific method and it requires specific training. *Education in mathematical modeling should focus more on developing skills on identifying biological problems which have a limited number of possible answers and which can be addressed using mathematical modeling*. For example, if one finds too many alternative explanations for his/her question, perhaps he/she is not asking a “good” question. In practice, consideration of two or more models would be likely to be better than study with a single model, and formulation and analysis of models with alternative core mechanisms is most preferable per strong inference.

It has to be realized that predictions of any single model for a biological system are not likely to be robust due to inherent openness of biological systems (Oreskes et al., 1994). Therefore, any single model is very limited in its use. However, a collection of alternative models is more likely to generate robust predictions; alternatively, analysis of such models could suggest inability to make robust predictions due to lack of appropriate data to reject alternative models. In this case, such multiple models-driven analysis may suggest areas for further experimental investigations. The idea of limited robustness of mathematical models in describing biological phenomena needs to be percolated in educational curriculum of undergraduate and graduate students, and this notion needs to be more widely stated in the professional modeling community. Realization that for every biological problem there are likely several alternative mechanisms/models needs to be eventually translated in research where it is not acceptable anymore to have a publication with only one mathematical model analyzed. We need to see mathematical biology research to move to the stage where in most publications the authors propose multiple models and discriminate between these models using quantitative biological data. *Education of future generation of mathematical modelers must include training in building of alternative mathematical models and in techniques to discriminate between alternative models using experimental data* (Burnham and Anderson, 2002; Johnson and Omland, 2004). When presented with results from a mathematical modeling-based study we should always ask **the question** (adapted from Platt, 1964): “But Sir/Madam, which mathematical models/mechanisms have you rejected in your study?”

*Training of a new generation of scientists in mathematical biology should involve more reading and discussion of the basics of scientific method*. Three papers are of particular importance and they should form the core of the graduate curriculum in graduate schools and specifically, of programs on mathematical modeling (Chamberlin, 1890; Platt, 1964; Oreskes et al., 1994). While I have discussed the ideas of the papers by Chamberlin (1890) and Platt (1964), an essay by Oreskes et al. (1994) clearly defined usefulness and limitations of mathematical modeling of open natural systems. In particular, the authors strongly cautioned against use of the words “verification” and “validation” to indicate “quality” of mathematical models as these terms exaggerate the limited ability of models to make robust predictions. In fact, “verification” of models is impossible per word definition due to the openness of natural systems, and in most cases the use of the word “validation” is synonymous to “verification” and thus is also inappropriate. The authors discussed in detail why verification/validations of models (or any logical statement) is impossible in natural sciences, and highlighted many philosophical developments on the nature of scientific method in the early Twentieth Century that is rarely discussed in graduate programs nowadays.

An important component of learning about mathematical modeling in biology is a realization that good modeling requires good understanding of the developed mathematical models. When does one understand the model, in a true sense of understanding? I believe that for simple models with a few parameters, true understanding is realized when one intuitively can predict the impact of the change in a model parameter or a combination of parameters on the model dynamics. Such an detailed understanding of the model also allows for insights in situations when the model is not able to fit/describe experimental data—i.e., why isn't the model able to explain experimental data? What is wrong with it? Deeper understanding of the model can point to parts of the model that are responsible for such discrepancy. Intuitive understanding of the model is very difficult or impossible for models with tens to hundreds of parameters. Yet, such an understanding is needed if the model fails to explain well some experimental data. How can one understand such a model? The traditional approach for understanding complex models is sensitivity analysis (Marino and Kirschner, 2004). Sensitivity analysis can allow to rank parameters of the model or the combination of parameters in terms of their impact on behavior of specific model components, e.g., density of species at some time point. I would argue, however, that in many cases sensitivity analyses do not give a good understanding of the model behavior because answers may depend on the method used and because sensitivity analysis often does not specify why this and not another parameter is the most important in the model dynamics. However, analyses which provide rational explanations of why specific parameters or parameter combinations drive model dynamics will likely reveal relative importance of different biological mechanisms. *Education of future mathematical modelers should include basics of sensitivity analyses and understanding when such analyses are informative and when they are not*.

## 5. Conclusions

A simple and effective critique of multiple hypotheses/models-driven research is to make counter examples of studies utilizing a single mathematical model and yet providing important biological insights. For instance, very well known studies utilizing a single ODE-based mathematical model estimated the rate of turnover of HIV and HIV-infected cells (Ho et al., 1995; Wei et al., 1995). Although the success of this pioneering work to accurately estimate the life-span of infected cells is well known, the failure of the model to accurately predict turnover of CD4 T cells due to incorrect assumption of CD4 T cell recovery due to production of new T cells is rarely acknowledged (Ho et al., 1995; Pabst and Rosenberg, 1998; Bucy et al., 1999). Furthermore, because we tend to remember “winners” and forget “losers,” it is very likely that many predictions of single mathematical modeling-based studies are incorrect or not robust to changes in the model assumption. It would be useful to generate data on the frequency of “correct" vs. “incorrect” predictions of studies based on single vs. multiple mathematical models although it may be difficult to define “correctness” of predictions.

Even in the absence of such data I propose that in order for mathematical modeling to become more robust, more practical and relevant for infectious disease biology we, mathematical modelers, need to re-think how we do research and how we train new generations of students. It is possible that the current format in which students, taking mathematical modeling in biology courses, get exposed to sets of standard models and their properties needs to be changed to observation-driven training where students develop models to explain particular experimental observations. Basic biological principles can be used to drive the development of models with variable levels of complexity and models the alternative mechanisms. Comparison to quantitative experimental data then can be used to test which of the models (i.e., mechanisms) are not consistent with the data and why (Popper, 2002).

Given that mathematical models are increasingly playing an important role in policy decision making (Christley et al., 2013), it is the time to change the way many mathematicians approach modeling, and we need to change the way we teach mathematical modeling at universities. Devising as many as possible alternative models for every biological question and comparing model predictions with quantitative experimental data to reject the models will allow mathematical modeling to become a scientific procedure generating more robust predictions.

## Author contributions

The author confirms being the sole contributor of this work and approved it for publication.

### Conflict of interest statement

The author declares that the research was conducted in the absence of any commercial or financial relationships that could be construed as a potential conflict of interest.
